# Editorial: Methods in cancer genetics

**DOI:** 10.3389/fonc.2023.1227854

**Published:** 2023-07-04

**Authors:** Simona D’Aguanno, Pawel Buczkowicz

**Affiliations:** ^1^ Preclinical Models and New Therapeutic Agents Unit, IRCCS Regina Elena National Cancer Institute, Rome, Italy; ^2^ PhenoTips, Toronto, ON, Canada

**Keywords:** cancer genetics, methods, PCR, WGS - whole-genome sequencing, ACMG, medical genetics, PDX (patient-derived xenograft), STR (short tandem repeat)

The evolution and innovation of methods used in cancer genetics research and their practical and clinical utility have drastically accelerated in the last few decades. The use of methods which have become part the standard toolkit of the genetics researcher, such as PCR, protein interaction assays and whole-genome sequencing (WGS) can be applied in a variety of novel ways in order to solve complex problems across the cancer genetics field. We collected five articles highlighting the applicability of novel methods in cancer genetics overlapping basic research, translational research, forensics and medical genetics.

PCR has long been a widely adopted method for quick and effective amplification of complete or partial segments of DNA, and the backbone for early DNA sequencing strategies such as Sanger (chain-termination) and sequencing by synthesis (SBS), and it continues to be critical for the powerhouse of genetics research, whole-genome sequencing. Many variations of this technology have been developed, beyond those used for sequencing and the applicability of PCR-based techniques includes interrogating copy number, gene expression, and methylation, among others. Two Original Research papers and one Methods paper in this Research Topic highlight the diversity and continued evolution of PCRs applicability in cancer genetics.

Cancer cell lines and xenograft models are an integral part of cancer genetics research. Instances of cell line contamination have been well documented, probably none more than that of many continuous cell lines contaminated with HELA cell. Furthermore, it is estimated that between one quarter to one-third of cell lines used by researchers are contaminated or misidentified (1). When harvesting tissue or cells from human-derived xenograft (PDX) models, one might expect some level of contamination between the human cells and that of the model organism. Jin et al. describe a method for a fast and accurate detection of interspecies contamination in PDX models and cell lines, and highlight a specific case of a human PDX mouse model transforming murine stromal cells into a malignant tumourigenic murine cell line, along with a detailed temporal analysis of the human to murine contamination. A highly sensitive intronic qPCR method was employed for detecting *GAPDH* intronic genomic copies in humans, and murine intronic *Gapdh* to assess the extent of contamination (Jin et al.). Eight human ascites-derived PDX models were tested post tumour excision and all tumours had varying degrees of murine cells, from as low as 4.72% to as high as 94.64%, which poses a problem for consistency and reliability of results when assessing the efficacy of *in vitro* drug experiments, and especially those for preclinical personalised therapies. One human ascites-derived PDX model described in the manuscript, from which tumour tissue was excised for the purpose of creating a patient-derived cell line, contained 28.08% murine cells. Three different subpopulations of cells were identified, both human and murine. Over time, *in vitro* passage of this cell line resulted in the purely murine cells that displayed greater *in vivo* tumourigenic properties than the human subpopulation. This rapid and sensitive qPCR-based method for detecting contamination of human PDX tumours has the potential to reduce the frequency of highly contaminated or misidentified human-derived xenograft models and the cell lines generated from them.

PCR-based methods can also be deployed for sensitive detection of somatic copy number variations in cancer. While methodologies for detecting oncogene amplification exist, the detection of somatic copy number loss is less frequently utilised due to lack of sensitivity or high cost of currently available methods. *CDKN2A* deletions are common in somatic cancer tissue and present clinically significant and actionable targets for therapies (2, 3). Tian et al. estimated common deletion regions (CDRs) in various tumour suppressor genes, including *CDKN2A*. The frequency of the 5.1kb CDR, which covers exon-2, was found in >90% of *CDKN2A*-deleted cancers. They subsequently developed a quantitative multiplex PCR assay P16-Light for the detection of somatic copy number loss in the gene and validated their findings using WGS (Tian et al.).

Long non-coding RNAs (lncRNAs) includes transcripts no longer than 200 nt, without specific protein coding functions. In the recent years, lncRNAs have been arousing more and more interest due to their potential application in the diagnosis of tumours and in the development of new therapies. Wang et al. used qRT-PCR to investigate lncRNAs expression in fresh colorectal cancer (CRC) tissues and adjacent tissues, finding 3,006 differentially expressed lncRNAs (Wang et al.). Among them, they focused on lncRNA 604, whose expression in CRC tissues was verified by FISH, finding higher lncRNA 604 expression in CRC tissues respect to normal tissues. Interestingly, low expression of lncRNA 604 was significantly associated to prolonged overall survival in CRC patients, respect to patients showing high expression of lncRNA 604. Biological function of lncRNA 604 has been investigated and *in vitro* experiments performed in different cell models of CRC demonstrated that lncRNA 604 promotes cell proliferation, migration and invasion. LncRNA 604 also inhibit AEG-1 by combining with miRNA564 in the cytoplasm and could regulate the nuclear transcription factor ZNF326 in the nucleus. *In vivo* experiments suggest lncRNA 604 promotes metastasis and chemoresistance.

PCR is but one technology that can be utilised for cancer genetics research. Repurposing of existing and established techniques, such Short Tandem Repeat (STR) identification from forensics was applied by Chen et al., along with next-generation sequencing (NGS) for tumour source identification (Chen et al.). To date, the STR status in tumours has been determined by capillary electrophoresis (CE). This approach allowed the classification of five STR statuses. Being heterogeneous, tumours are composed of a mixture of cells with different STR statuses, which need a method of detection sensitive enough to allow for profiling and discrimination of those differences. Thus, the authors employed NGS, known to be a highly sensitive application for tumour source identification. In this paper, both CE and a general recognised method of NGS have been employed to profile a total of 55 paired tumour samples, including different tumour histotypes and peripheral blood samples from 75 subjects. Comparing the obtained results, the authors observed a concordance of 91.43%. between the two approaches. The authors also generated a more sensitive NGS method for tumour source identification, helpful to identify more germline-originated alleles.

Ultimately, all cancer genetics research aims to positively impact diagnosis, patient care and clinical decision making. Careca and Radice implemented assays based on reassembly of Green Fluorescent Protein (GFP to assess the effects of sequence mutation of *BRAC1* classified as variants with uncertain significance (VUS) in breast cancer (BC) (Careca and Radice). The risk of developing BC is cumulatively increased with the presence of the so-called germline pathogenic variants (PVs) in the *BRCA1* and *BRCA2* genes commonly recognised as tumour suppressor genes. The usefulness of sequencing *BRCA1* and *BRCA2* is often limited by the occurrence of VUS, whose protein function and clinical relevance are unknown. In this paper, Careca and Radice aimed to generate an experimental approach useful to characterise the function of eight selected variants localised to the RING finger and BRCT domains of *BRCA1*. Performing *in vitro* GFP-reassembly screening they evaluated how these variants could modify the binding of the RING finger and BRCT domains with UbcH5a or ABRAXAS, respectively. In order to verify whetherBRCA1-ABRAXAS binding assay was able to correctly discriminate among pathogenic and non-pathogenic variants, the authors analysed a panel of variants classified according to the IARC 5-class model (Leiden Open Variation Database, URL: http://hci-exlovd.hci.utah.edu/variants.php) (4). Subsequently, Careca and Radice used *in vivo* semi-quantitative Mammalian Two-Hybrid approach to validate the data obtained from the GFP-reassembly screening. Finally, the authors combined the results of their assays with those described in the “Hi Set” study (5), clarifying the functional significance of *BRCA1* VUS and on their clinical interpretation within the ACMG/AMP framework (6).

These five manuscripts describing methods provide an example of how novel applications of tools can be used by researchers and clinicians to interrogate cancer tissues, cell lines and models in the field of cancer genetics.

## Author contributions

Writing, review, and editing: SD and PB. All authors contributed to the article and approved the submitted version.
